# Prognostic Value of Systemic Inflammatory Indices, NLR, PLR, and MPV, for Predicting 1-Year Survival of Patients Undergoing Cytoreductive Surgery with HIPEC

**DOI:** 10.3390/jcm8050589

**Published:** 2019-04-29

**Authors:** Na Young Kim, Duk-Hee Chun, So Yeon Kim, Nam Kyu Kim, Seung Hyuk Baik, Jung Hwa Hong, Kyung Sub Kim, Cheung-soo Shin

**Affiliations:** 1 Department of Anesthesiology and Pain Medicine, Anesthesia and Pain Research Institute, Severance Hospital, Yonsei University College of Medicine, 50-1 Yonsei-ro, Seodaemun-gu, Seoul 03722, Korea; knnyyy@yuhs.ac (N.Y.K.); KIMSY326@yuhs.ac (S.Y.K.); 2 Department of Anesthesiology and Pain Medicine, CHA Bundang Medical Center, CHA University school of Medicine, 59 Yatap-ro, Seongnam, Gyeonggi-do 13496, Korea; leah1013@chamc.co.kr; 3 The Division of Colon and Rectal Surgery, Department of Surgery, Severance Hospital, Yonsei University College of Medicine, 50-1 Yonsei-ro, Seodaemun-gu, Seoul 03722, Korea; NAMKYUK@yuhs.ac; 4 The Division of Colon and Rectal Surgery, Department of Surgery, Gangnam Severance Hospital, Yonsei University College of Medicine, 211 Eonju-ro, Gangnam-gu, Seoul 06273, Korea; WHITENOJA@yuhs.ac; 5 Department of Policy Research Affairs National Health Insurance Service Ilsan Hospital, 100 Ilsan-ro, Ilsandong-gu, Goyang, Gyeonggi-do 10444, Korea; jh_hong@nhimc.co.kr; 6 Department of Anesthesiology and Pain Medicine, Anesthesia and Pain Research Institute, Gangnam Severance Hospital, Yonsei University College of Medicine, 211 Eonju-ro, Gangnam-gu, Seoul 06273, Korea; SYBER1992@yuhs.ac

**Keywords:** neutrophil to lymphocyte ratio, platelet to lymphocyte ratio, mean platelet volume, hyperthermic intraperitoneal chemotherapy

## Abstract

The neutrophil to lymphocyte ratio (NLR), platelet to lymphocyte ratio (PLR), and mean platelet volume (MPV) have been reported to be associated with the prognosis of various types of tumors. This study evaluated the prognostic value and clinical use of inflammatory markers for predicting 1-year survival in patients undergoing cytoreductive surgery (CRS) with hyperthermic intraperitoneal chemotherapy (HIPEC). This retrospective study included 160 patients who underwent CRS with HIPEC between July 2014 and April 2017. Data on NLR, PLR, and MPV were collected preoperatively and on postoperative days (POD) 1, 2, 3, 4, and 5. In a multivariate analysis using a cox proportional hazard regression model, higher values of preoperative NLR and MPV, PLR, and MPV on POD 2, 3, and 5 were associated with reduced 1-year survival after CRS with HIPEC. Patients with increased MPV showed lower rates of 1-year survival following CRS with HIPEC. In addition, elevated preoperative NLR and postoperative PLR were correlated with poor survival. These markers are able to stratify patients by risk profile, which may ultimately improve perioperative management and be helpful in improving outcomes following CRS with HIPEC.

## 1. Introduction

Peritoneal invasion of tumors is generally regarded as a terminal cancer stage, which has very poor survival outcomes indicating metastasis of a primary cancer into the peritoneum [1]. For treatment of patients with peritoneal carcinomatosis, cytoreductive surgery (CRS) with hyperthermic intraperitoneal chemotherapy (HIPEC) has become a promising strategy to improve survival benefits [2,3,4,5,6,7]. For such advanced cancer patients undergoing CRS with HIPEC, easily measured and accurate prognostic biomarkers are needed to predict the course of postoperative outcomes and even guide treatment.

Inflammation plays an important role in the development of cancer in that inflammatory conditions augment carcinogenesis and inflammatory mediators produced by tumor cells further promote the development of tumors [8]. This imbalance in the immune system due to excessive inflammatory responses promotes tumor cell growth and can lead to poor survival outcomes. Thus, many studies have investigated the ability of inflammatory biomarkers to act as potential prognostic predictors capable of demonstrating the inflammatory status in various types of cancers [9,10,11,12,13].

Changes in the number and composition of circulating cells in the blood are related to systemic inflammation, and in the process of inflammatory regulation, neutrophils, lymphocytes, and platelets are important mediators of inflammation [14]. Of note, measurement of the neutrophil to lymphocyte ratio (NLR), platelet to lymphocyte ratio (PLR), and mean platelet volume (MPV) is an inexpensive approach; further, these values are easily evaluated in most hospital laboratories, and these markers have been reported to be significantly associated with the prognosis of cancers including peritoneal carcinomatosis [9,10,11,12,13,15,16]. To the best of our knowledge, few studies have reported on the prognostic value of the NLR, PLR, and MPV for peritoneal carcinomatosis in patients undergoing CRS combined with HIPEC, and a degree of controversy remains on the subject.

Hence, we evaluated the prognostic value and clinical use of inflammatory biomarkers, including the NLR, PLR, and MPV, for predicting 1-year survival in patients undergoing CRS combined with HIPEC and compared their prognostic abilities.

## 2. Material and Methods

### 2.1. Patients

**Figure 1:**
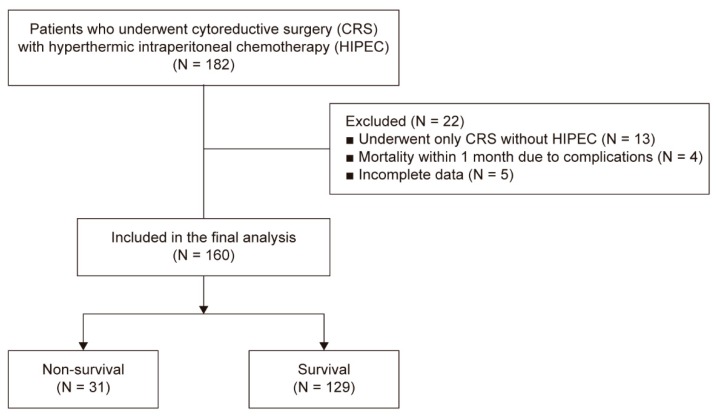
Flow diagram of patients.

This retrospective study included the electronic medical records acquired from two tertiary referral hospitals (Severance Hospital and Gangnam Severance Hospital) of the Yonsei University Health Systems (Seoul, Korea). Following approval by the Institutional Review Board and Hospital Research Ethics Committee (Yonsei University Health System, Seoul, Korea; IRB protocol No. 4-2017-0187), waiver of informed consent was granted because of the retrospective nature of the study. The data of 182 patients who underwent CRS and HIPEC from July 2014 to April 2017 were reviewed. Among these 182 patients, 22 were excluded for the following reasons: 13 patients underwent only CRS without HIPEC, 4 patients died due to complications within 1 month, and 5 patients had incomplete data (Figure 1).

### 2.2. Data Collection

All data were retrospectively collected from the electronic medical records. The demographics and perioperative variables included age, sex, body mass index (BMI), American Society of Anesthesiologists (ASA) physical status, underlying diseases such as hypertension and diabetes, prior chemotherapy, and preoperative peritoneal cancer index (PCI) scores. Operative data included the combined surgery, operation time, intraoperative fluid input and output, number of patients who received inotropic agents such as phenylephrine and norepinephrine, and perioperative temperatures. The number of patients who were admitted to the intensive care unit (ICU) and Acute Physiology and Chronic Health Evaluation scores at ICU admission were also collected. The NLR, PLR, and MPV were collected preoperatively (Preop) and on postoperative days (POD) 1, 2, 3, 4, and 5.

### 2.3. Statistical Analysis

Continuous variables are described as frequency or mean (standard deviation) and categorical variables are described as number of patients (percentage). Continuous variables were compared by using the independent *t*-test, and categorical variables were compared by using the chi-square test or Fisher’s exact test.

A linear mixed model was used to evaluate changes in the mean values of the NLR, PLR, and MPV over time. Two fixed effects were included: One between-subjects effect to assess the group effect (non-survival or survival) and one within-subjects effect to assess the time effect (Preop, PODs 2, 3, 4, and 5). A possible difference in sequence from Preop to POD 5 was analyzed using the group × time interaction. The group × time interaction was tested with a significance level of 0.05.

Univariate analysis was conducted to evaluate the independent factors for 1-year survival. Multivariate Cox proportional hazard regression was performed to identify associated independent factors, and the results are presented as hazard ratios (HRs) and 95% confidence intervals (CIs).

The Contal and O’Quigley method, which selects an optimal cutoff point by maximizing the HR, was selected at an optimal cutoff point by maximizing the HR and was performed to predict the optimal cutoff values based on time-to-event for dichotomization of clinical outcome variables. Kaplan-Meier curves were created based on 1-year survival, and the groups were compared by using the log-rank test. SAS version 9.4 (SAS Institute Inc., Cary, NC, USA) was used for all statistical analyses. A *p*-value < 0.05 was considered statistically significant.

## 3. Results

### 3.1. Demographics and Perioperative Variables

The demographics and perioperative variables of the 160 patients with peritoneal carcinomatosis who underwent CRS with HIPEC are provided in Table 1. The BMI in the non-survival group was significantly lower than that in the survival group. The non-survival group also had significantly higher scores of preoperative PCI and received more packed red blood cells (pRBC) intraoperatively than did the survival group. There were no significant differences in other variables between the non-survival and survival groups.

### 3.2. Systemic Inflammatory Indexes, Based on the NLR, PLR, and MPV

**Figure 2:**
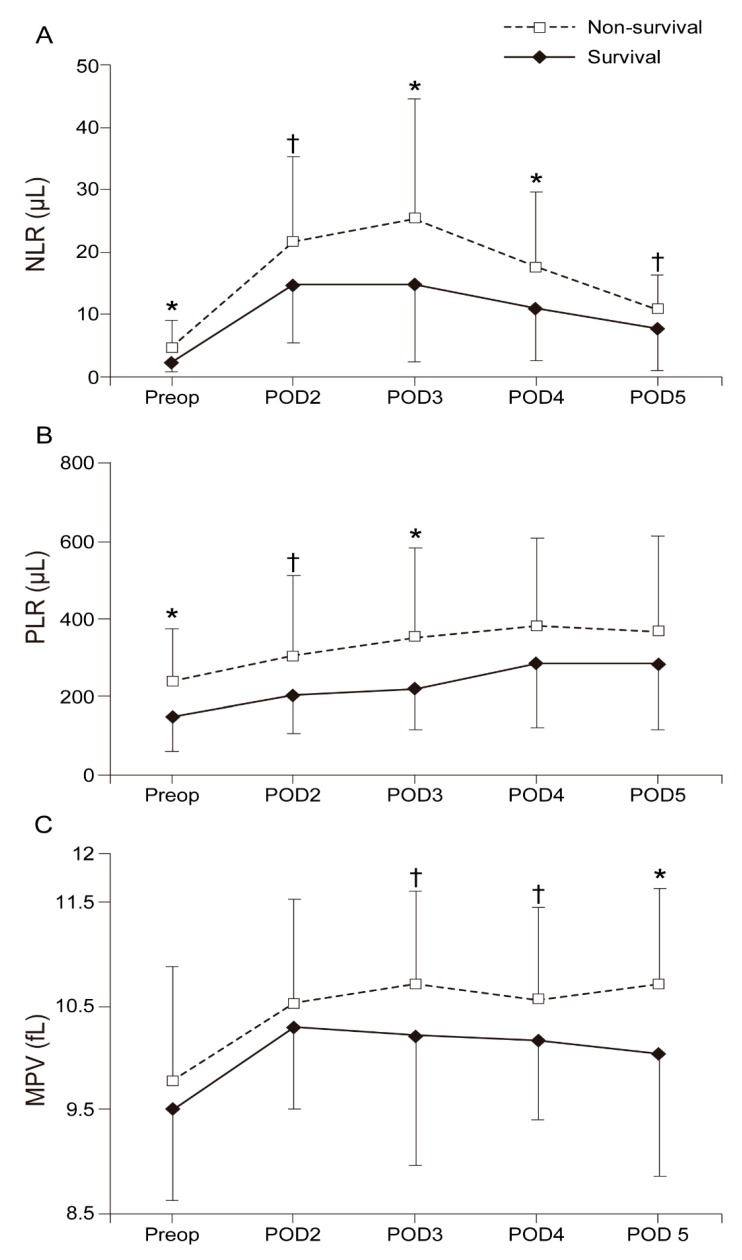
Changes in the mean value of the (**A)** NLR, (**B**) PLR, and (**C**) MPV from Preop until POD 5. NLR—neutrophil to lymphocyte ratio; PLR—platelet to lymphocyte ratio; MPV—mean platelet volume; Pre-op—preoperative; POD 2—2 days after surgery; POD 3—3 days after surgery; POD 4—4 days after surgery; POD 5—5 days after surgery. * *p* < 0.05 versus Survival. † *p* < 0.1 versus Survival.

Figure 2 shows the changes in the mean values of the NLR, PLR, and MPV from Preop until POD 5. The NLRs in the survival group were significantly lower than those in the non-survival group in Preop (2.2 (1.5) vs. 4.6 (4.4); Bonferroni corrected *p* = 0.025), on POD 3 (14.8 (12.5) vs. 25.3 (19.4); Bonferroni corrected *P* = 0.039), and on POD 4 (10.9 (8.5) vs. 17.5 (12.0); Bonferroni corrected *p* = 0.038). Significantly lower PLRs were observed in the survival group than in the non-survival group in Preop (150.7 (84.8) vs. 243.3 (135.8); Bonferroni corrected *p* = 0.005) and on POD 3 (226.7 (107.3) vs. 358.2 (228.5); Bonferroni corrected *p* = 0.022). The MPVs in the survival group were significantly lower than those in the non-survival group on POD 5 (10.1 (1.2) vs. 10.7 (0.9); Bonferroni corrected *p* = 0.037).

### 3.3. Prognostic Factors Affecting 1-Year Survival after CRS with HIPEC

Table 2 shows the prognostic factors affecting 1-year survival based on the univariate analysis. BMI, preoperative PCI scores, amounts of administered pRBC, the values of NLR and PLR from Preop until POD 5, and the values of MPV on PODs 3, 4, and 5 significantly affected 1-year survival. A multivariate analysis using a Cox proportional hazard regression model was performed at each time point and showed that higher values of preoperative NLR and MPV, PLR, and MPV on PODs 2, 3, and 5 were associated with reduced 1-year survival after CRS with HIPEC (Table 3).

**Figure 3:**
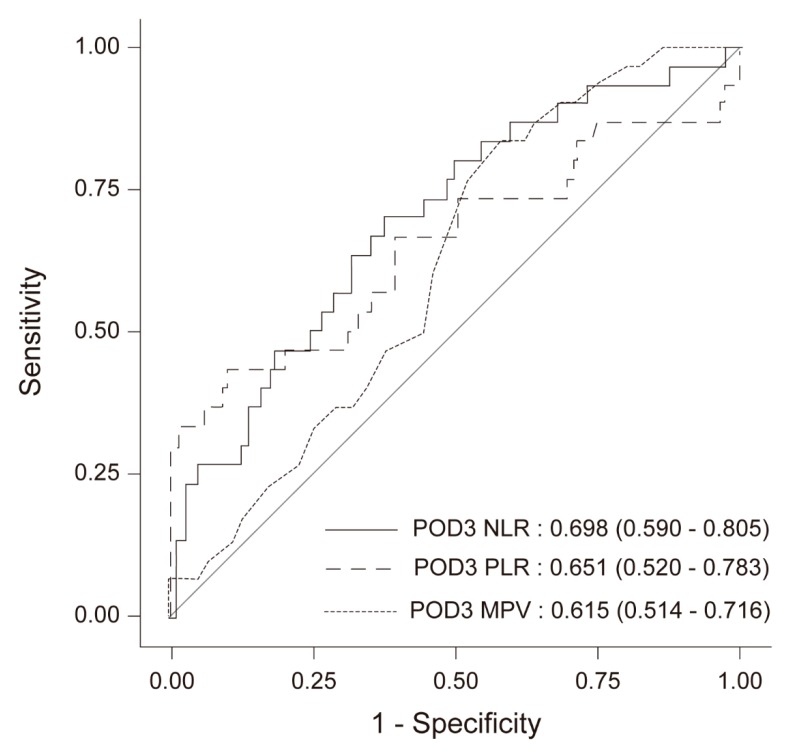
ROC curves of the NLR, PLR, and MPV on POD 3 based on the multivariate logistic regression. NLR—neutrophil to lymphocyte ratio; PLR—platelet to lymphocyte ratio; MPV—mean platelet volume; POD 3—3 days after surgery; ROC—receiver operating characteristics.

Figure 3 presents the receiver operating characteristic (ROC) curves of NLR, PLR, and MPV based on the multivariate logistic regression on POD 3. The area under the ROC curve for NLR, PLR, and MPV was 0.698 (95% CI 0.590–0.805), 0.651 (95% CI 0.520–0.783), and 0.651 (95% CI 0.514–0.716), respectively.

### 3.4. Overall Survival

**Figure 4:**
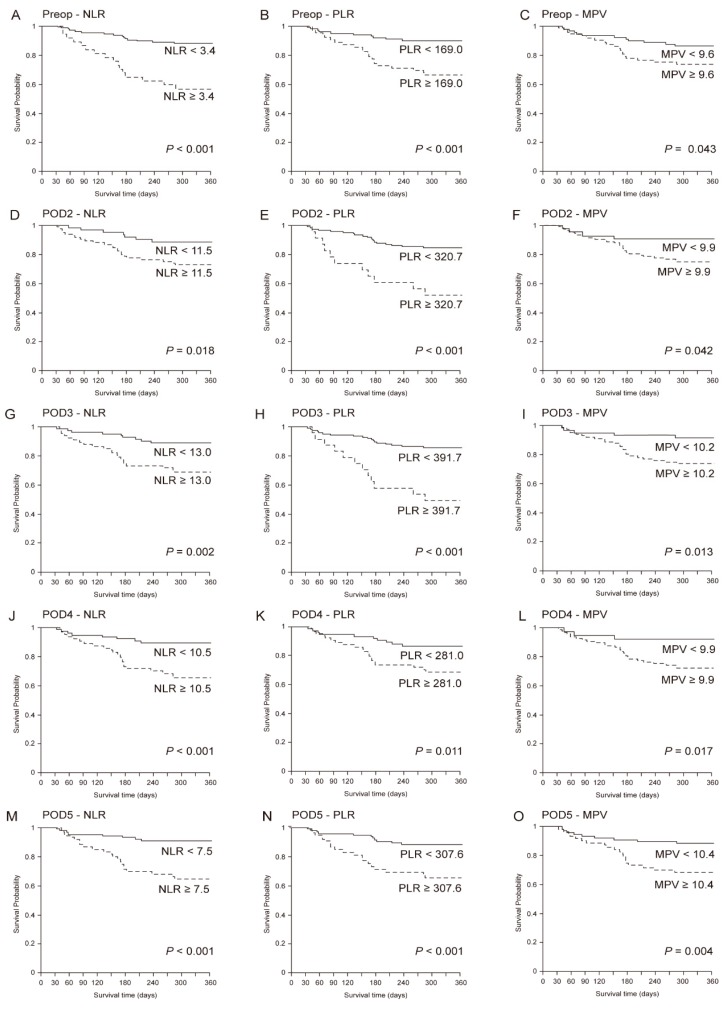
Kaplan-Meier curves of each variable (NLR, PLR, and MPV) from Preop until POD 5 according to the cutoff values based on the log-rank tests. (**A**) Preop-NLR, (**B**) Preop-PLR, (**C**) Preop-MPV. (**D**) POD2-NLR, (**E**) POD2-PLR, (**F**) POD2-MPV, (**G**) POD3-NLR, (**H**) POD3-PLR, (**I**) POD3-MPV, (**J**) POD4-NLR, (**K**) POD4-PLR, (**L**) POD4-MPV, (**M**) POD5-NLR, (**N**) POD5-PLR, (**O**) POD5-MPV. NLR—neutrophil to lymphocyte ratio; PLR—platelet to lymphocyte ratio; MPV—mean platelet volume; Pre-op—preoperative; POD 2—2 days after surgery; POD 3—3 days after surgery; POD 4—4 days after surgery; POD 5—5 days after surgery.

The mean follow-up time in all patients was 45.0 (25.2) months, with a mean disease-free time of 639.7 (249.6) days in the survival group and 137.8 (73.0) days in the non-survival group (*p* < 0.001). Figure 4 shows the Kaplan-Meier curve of each variable (NLR, PLR, and MPV) from Preop until POD 5 according to the cutoff values based on the log-rank tests. The results from the Contal and O’Quigley method, Kaplan-Meier analyses, and log-rank tests showed that there was a correlation between decreased 1-year survival and higher NLR, PLR, and MPV from preop until POD 5.

## 4. Discussion

In the current study, we evaluated the prognostic value of the NLR, PLR, and MPV for predicting 1-year survival in patients undergoing CRS combined with HIPEC. The main finding was that patients with increased MPV showed lower rates of 1-year survival. In addition, elevated preoperative NLR and postoperative PLR were correlated with poor survival.

There have been increasing reports on the association between systemic inflammation and poor prognosis in various types of cancer [10,17,18]. Recently, inflammatory biomarkers have become a useful tool for risk stratification of prognosis and overall survival in cancer patients [19,20,21,22,23]. However, among various types of malignancies, patients with carcinomatosis who underwent CRS with HIPEC are assumed to be at the advanced stage, which represents high aggressiveness and maximum host inflammation. To date, only few studies have been performed in patients undergoing CRS with HIPEC; thus, it is still controversial whether inflammatory biomarkers may be helpful prognostic predictors in such advanced-stage cancer patients.

The NLR, PLR, and MPV have been evaluated as novel inflammation markers in many cancer types since the tests for these markers are inexpensive and are easily performed using routine laboratory analysis [15]. The NLR is calculated by dividing the number of neutrophils by the number of lymphocytes, which shows the relative difference of the neutrophil and lymphocyte counts, and the PLR is calculated as the number of platelets divided by the lymphocyte count, which presents the relative difference of the platelet and lymphocyte counts [24]. Recent studies have shown the utility of the NLR as a prognostic tool to predict disease-free and overall survivals in cancer patients [25,26,27,28]. However, few studies have been performed on the preoperative NLR and survival in patients undergoing CRS with HIPEC [15]. Consistent with previous report, the multivariate analysis in the present study showed that elevated preoperative NLR was an independent prognostic factor for 1-year survival in patients undergoing CRS with HIPEC [15]. As with the NLR, the PLR has been investigated by numerous studies and identified as an independent risk factor for predicting survival in cancer patients [9,10,12,29]. A meta-analysis involving 12,754 patients determined the PLR as an independent prognostic marker associated with overall survival in solid tumors [30], and Bong et al. [16]. In the present study, however, following multivariate analysis, there was no significant difference in the preoperative PLR. This discrepancy was probably caused by the fact that the preoperative PLR was <300 in both the non-survival and survival groups; thus, the preoperative PLR was found to be a significant factor only in the univariate analysis. However, the multivariate analysis showed that the postoperative PLRs were associated with worsened 1-year survival in the present study.

The MPV is the mean size of platelets in the serum and reflects the rate and stimulation of platelet production [31]; it is regularly measured using blood count analyzers. Although the role of MPV levels in inflammatory process responses has been investigated in the literature, it remains less studied compared with NLR and PLR [31,32,33]. High-volume platelets have been reported to be critically involved in the infiltration of inflamed tissue [34]. In addition, high-volume platelet infiltration has been shown to be a sign of inflammation in hepatocellular carcinoma, pancreatic adenocarcinoma, endometrial cancer, lung cancer, and gastric cancer [35,36,37]. Thus far, there have been no studies on MPV and postoperative prognosis in patients undergoing CRS with HIPEC. This retrospective study was the first to investigate the potential association between MPV and postoperative prognosis in peritoneal carcinomatosis patients who underwent CRS with HIPEC. An elevated preoperative MPV showed the most potent hazard ratio (HR) in predicting postoperative prognosis (HR, 1.658 (95% CI, 1.071–2.566)) compared to preoperative NLR or PLR after multivariate analysis. Furthermore, increased MPV values were significantly associated with prognosis both preoperatively and postoperatively. Elevated MPVs on PODs 2, 3, and 5 were independently associated with poor 1-year survival, with postoperative HR following an increasing trend (HR = 1.522, 1.820, and 1.970, respectively). This finding is similar to the findings of a previous study with colon cancer patients with stages III–IV who had significantly higher levels of MPV compared to the patients with stages I–II, and a significant reduction in MPV levels was found after surgical tumor resection [38]. Therefore, postoperative MPV increases reflect the aggressiveness of the tumor and inflammation, thus confirming their prognostic capabilities.

There are some limitations to this study. First, this study had a two-center design and was retrospective in nature; thus, it was difficult to confirm that the results obtained were completely representative of all patients undergoing CRS with HIPEC. Second, additional multiple confounding factors may have also affected the NLR, PLR, and MPV, but were not controlled for in the current study. Third, patients who underwent CRS with HIPEC due to ovarian cancers were not included in this study; thus, the outcome of those patients cannot be estimated based on the present results. Finally, a small sample size was used in the present study and patients were only followed up for a short period. Due to these restrictions, future research should focus on recruiting a larger number of patients and using longer follow-up periods to evaluate long-term survival rates. Nevertheless, clinically valuable points were found in this retrospective study. To the best of our knowledge, this study was the first to demonstrate the combined utilities of the NLR, PLR, and MPV from the preoperative to the postoperative period for determining the prognosis of patients who underwent CRS with HIPEC. This study provided evidence for a relationship between these inflammatory biomarkers and survival in patients with carcinomatosis undergoing CRS with HIPEC; thus, they may be appropriate biomarkers for use in the assessment of treatment efficacy to decide further treatment allocation.

In summary, patients with increased MPV showed a lower rate of 1-year survival following CRS with HIPEC. In addition, elevated preoperative NLR and postoperative PLR were also correlated with poor survival. These markers may be used to stratify patients by risk profile to ultimately augment perioperative management and may be helpful to improve outcomes following CRS with HIPEC.

## Figures and Tables

**Table 1:** Demographics and perioperative variables.

Variables	Non-Survival (*N* = 31)	Survival (*N* = 129)	*p* Value
Age, years	55.7 (13.5)	55.1 (11.8)	0.815
Male sex	19 (61%)	60 (47%)	0.140
Body mass index, kg/m^2^	19.9 (3.7)	22.0 (3.7)	0.007 *
ASA physical status			0.285
I	5 (16%)	37 (29%)	
II	20 (65%)	76 (59%)	
III	6 (19%)	16 (12%)	
Comorbidities			
Hypertension	7 (23%)	37 (29%)	0.495
Diabetes mellitus	2 (6%)	18 (14%)	0.369
Prior chemotherapy	24 (77%)	81 (63%)	0.124
Preoperative PCI score	19.4 (11.7)	12.6 (10.9)	0.004 *
Combined surgery	20 (65%)	78 (60%)	0.678
Operation time, min	649.5 (191.3)	593.2 (195.3)	0.150
Intraoperative input and output			
Total fluid, mL	7183 (2694)	6900 (3168)	0.647
Colloid, mL	952 (582)	6032 (2960)	0.425
20% Albumin, mL	148 (190)	111 (160)	0.259
Blood loss, mL	1373 (978)	1076 (1028)	0.148
Urine output, mL	1040 (670)	1104 (718)	0.652
pRBC, *n*	1.9 (2.4)	0.9 (1.5)	0.039 *
Number of patients who received inotropic agents			
Phenylephrine, *n*	13 (42%)	66 (51%)	0.356
Norepinephrine, *n*	20 (65%)	60 (47%)	0.072
Temperature			
Prior to operation	36.3 (0.5)	36.2 (0.3)	0.161
Highest	38.6 (0.6)	38.5 (0.8)	0.430
At the end of operation	36.3 (0.5)	36.4 (0.6)	0.621
ICU admission, *n*	30 (97%)	112 (87%)	0.202
APACHE score	16.4 (6.6)	15.1 (6.8)	0.346
Types of cancer			0.425
Appendiceal cancer	6 (19.4%)	23 (17.8%)	
Ascending colon cancer	7 (22.6%)	21 (16.3%)	
Cecal cancer	3 (9.7%)	6 (4.7%)	
Descending colon cancer	2 (6.5%)	3 (2.3%)	
Peritoneal mesothelioma	1 (3.2%)	7 (5.4%)	
Rectal cancer	5 (16.1%)	19 (14.7%)	
Sigmoid cancer	5 (16.1%)	43 (33.3%)	
Transverse colon cancer	2 (6.5%)	7 (5.4%)	
Preoperative			
NLR	4.6 (4.4)	2.2 (1.5)	0.005 *
PLR	243.3 (135.8)	150.7 (84.8)	<0.001 *
MPV	9.8 (1.1)	9.5 (0.9)	0.094
Postoperative Day 2			
NLR	21.6 (13.7)	14.7 (9.2)	0.014 *
PLR	309.3 (208.6)	207.0 (95.3)	0.013 *
MPV	10.6 (1.0)	10.3 (0.8)	0.153
Postoperative Day 3			
NLR	25.3 (19.4)	14.8 (12.5)	0.008 *
PLR	358.2 (228.5)	226.7 (107.3)	0.004 *
MPV	10.7 (0.9)	10.2 (1.3)	0.018 *
Postoperative Day 4			
NLR	17.5 (12.0)	10.9 (8.5)	0.008 *
PLR	385.3 (229.1)	291.7 (166.0)	0.043 *
MPV	10.6 (0.9)	10.2 (0.8)	0.013 *
Postoperative Day 5			
NLR	10.8 (5.5)	7.6 (6.7)	0.019 *
PLR	373.2 (246.2)	290.0 (167.5)	0.094 *
MPV	10.7 (0.9)	10.1 (1.2)	0.007 *

Values are presented as mean (standard deviation) or number of patients (%). * *p* < 0.05. APACHE—Acute Physiology and Chronic Health Evaluation; ASA—American Society of Anesthesiologists; PCI—peritoneal cancer index; pRBC—packed red blood cell; ICU—intensive care unit; NLR—neutrophil to lymphocyte ratio; PLR—platelet to lymphocyte ratio; MPV—mean platelet volume.

**Table 2:** Univariate analyses for prognostic factors for 1-year survival after CRS with HIPEC.

Variables	1-Year Survival	
	HR (95% CI)	*p* Value
Age, years	1.005 (0.976–1.035)	0.750
Female sex	1.691 (0.821–3.485)	0.154
Body mass index, kg/m^2^	0.872 (0.787–0.966)	0.009*
ASA physical status		
I	1	
II	1.804 (0.677–4.806)	0.238
III	2.513 (0.767–8.236)	0.128
Comorbidities		
Hypertension	0.762 (0.328–1.769)	0.527
Diabetes mellitus	0.448 (0.107–1.876)	0.272
Prior chemotherapy	1.934 (0.833–4.488)	0.125
Preoperative PCI score	1.042 (1.012–1.074)	0.006 *
Combined surgery	1.144 (0.548–2.388)	0.720
Operation time, min	1.001 (0.999–1.003)	1.956
Intraoperative input and output		
Total fluid, mL	1.000 (1.000–1.000)	0.769
Colloid, mL	1.000 (1.000–1.001)	0.470
20% Albumin, mL	1.112 (0.924–1.338)	0.261
Blood loss, mL	1.000 (1.000–1.000)	0.179
Urine output, mL	1.000 (0.999–1.000)	0.589
pRBC, *n*	1.226 (1.057–1.421)	0.007 *
Number of patients who received inotropic agents		
Phenylephrine, *n*	0.717 (0.351–1.464)	0.361
Norepinephrine, *n*	1.972 (0.945–4115)	0.071
Temperature		
Prior to operation	2.316 (0.895–5.991)	0.083
Highest	1.217 (0.760–1.950)	0.414
At the end of operation	0.852 (0.451–1.610)	0.622
ICU admission, *n*	4.177 (0.570–30.629)	0.160
APACHE score	1.024 (0.975–1.076)	0.340
Types of cancer		
Appendiceal cancer	1	
Ascending colon cancer	1.194 (0.401–3.552)	0.750
Cecal cancer	1.558 (0.389–6.229)	0.531
Descending colon cancer	2.381 (0.480–11.812)	0.288
Peritoneal mesothelioma	0.529 (0.064–4.398)	0.529
Rectal cancer	1.037 (0.316–3.397)	0.952
Sigmoid cancer	0.463 (0.141–1.518)	0.204
Transverse colon cancer	1.029 (0.208–5.097)	0.972
Preoperative		
NLR	1.289 (1.185–1.403)	<0.001 *
PLR	1.005 (1.003–1.007)	<0.001 *
MPV	1.371 (0.942–1.993)	0.099
Postoperative day 2		
NLR	1.037 (1.014–1.061)	0.001 *
PLR	1.004 (1.002–1.006)	<0.001 *
MPV	1.316 (0.895–1.935)	0.163
Postoperative day 3		
NLR	1.026 (1.011–1.041)	<0.001 *
PLR	1.005 (1.003–1.007)	<0.001 *
MPV	1.564 (1.062–2.303)	0.023 *
Postoperative day 4		
NLR	1.041 (1.016–1.067)	0.001 *
PLR	1.002 (1.000–1.004)	0.012 *
MPV	1.617 (1.099–2.381)	0.015 *
Postoperative day 5		
NLR	1.038 (1.005–1.073)	0.025 *
PLR	1.002 (1.000–1.004)	0.018 *
MPV	1.884 (1.312–2.707)	<0.001 *

Values are presented as hazard ratio (95% confidence interval). * *p* < 0.05 APACHE—Acute Physiology and Chronic Health Evaluation; ASA—American Society of Anesthesiologists; CI—confidence interval; HR—hazard ratio; PCI—peritoneal cancer index; pRBC—packed red blood cell; ICU—intensive care unit; NLR—neutrophil to lymphocyte ratio; PLR—platelet to lymphocyte ratio; MPV—mean platelet volume.

**Table 3:** Multivariate analyses for independent factors associated with increased risk of 1-year survival at each time point.

	Preoperative		Postoperative Day 2		Postoperative Day 3		Postoperative Day 4		Postoperative Day 5	
	HR (95% CI)	*p* Value	HR (95% CI)	*p* Value	HR (95% CI)	*p* Value	HR (95% CI)	*p* Value	HR (95% CI)	*p* Value
BMI	0.897 (0.789–1.021)	0.099	0.968 (0.856–1.094)	0.600	0.978 (0.866–1.105)	0.719	0.961 (0.853–1.083)	0.516	0.948 (0.840–1.069)	0.384
Pre-PCI score	1.016 (0.980–1.054)	0.375	1.017 (0.981–1.055)	0.359	1.015 (0.977–1.054)	0.436	1.025 (0.987–1.064)	0.204	1.029 (0.993–1.066)	0.113
pRBC, *n*	1.180 (0.952–1.463)	0.130	1.216 (0.968–1.528)	0.092	1.192 (0.970–1.466)	0.095	1.112 (0.899–1.376)	0.326	1.194 (0.958–1.489)	0.115
NLR	1.250 (1.118–1.398)	<0.001 *	0.983 (0.944–1.024)	0.420	0.977 (0.953–1.002)	0.071	0.992 (0.954–1.032)	0.705	0.958 (0.906–1.013)	0.135
PLR	1.001 (0.998–1.004)	0.482	1.004 (1.002–1.007)	0.001 *	1.005 (1.003–1.008)	<0.001 *	1.002 (1.000–1.003)	0.115	1.003 (1.001–1.005)	0.007 *
MPV	1.658 (1.071–2.566)	0.023 *	1.522 (1.017–2.276)	0.041 *	1.820 (1.158–2.859)	0.009 *	1.532 (0.978–2.398)	0.062	1.970 (1.298–2.990)	0.002 *

Values are presented as the hazard ratio (95% confidence interval). * *p* < 0.05. BMI—body mass index; CI—confidence interval; HR—hazard ratio; Pre-PCI—preoperative peritoneal cancer index; pRBC—packed red blood cell; NLR—neutrophil to lymphocyte ratio; PLR—platelet to lymphocyte ratio; MPV—mean platelet volume.
